# Supplementary material for: Eriocheir sinensis feminization-1c (Fem-1c) and Its Predicted miRNAs Involved in Sexual Development and Regulation
Source: Animals (Basel). 2023 May 30;13(11):1813. doi: 10.3390/ani13111813 (PMC10251896; doi:10.3390/ani13111813)
Supplement: Supplementary file 1 [file animals-13-01813-s001.zip › Figures S1-S3.pdf]

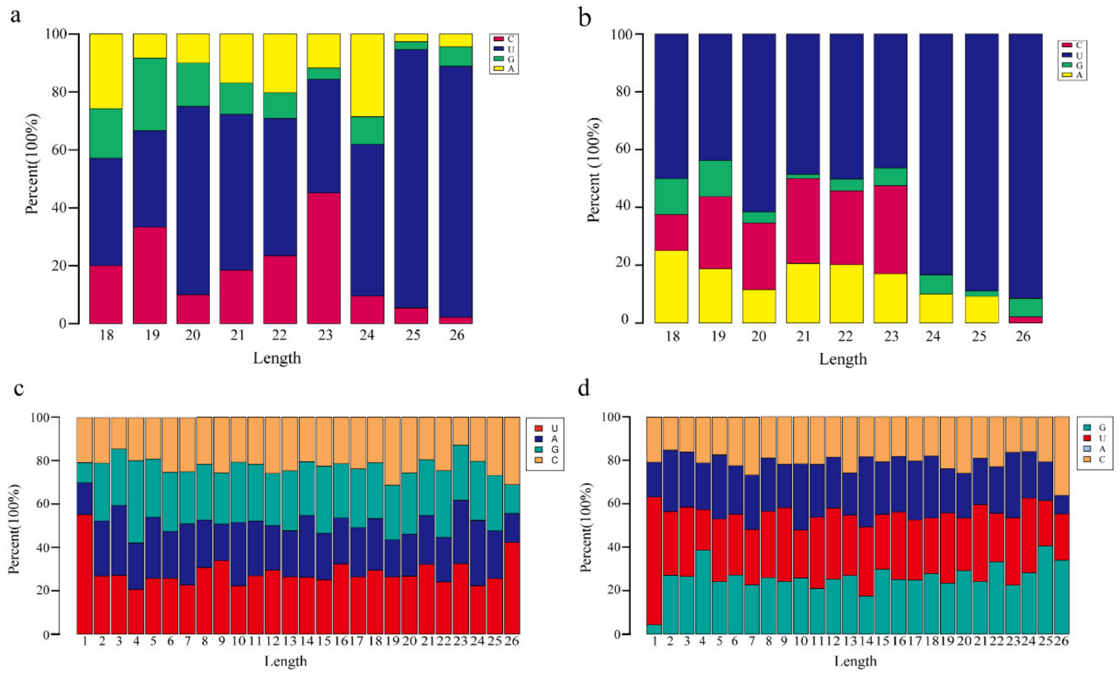

Figure S1. The nucleotide bias of known miRNAs at first and each position in female and male crabs. (**a**) and (**b**) The nucleotide bias of miRNAs at the first position in female and male crabs. The X-axis represented the base position of miRNA, while the Y-axis represented the percentage of A/U/C/G bases in the miRNA at the first position. (**c**) and (**d**) The nucleotide bias of miRNAs from female and male crabs at each position. The X-axis represented the base position of the miRNA, while the Y-axis represented the percentage of A/U/C/G bases in the miRNA at each position.

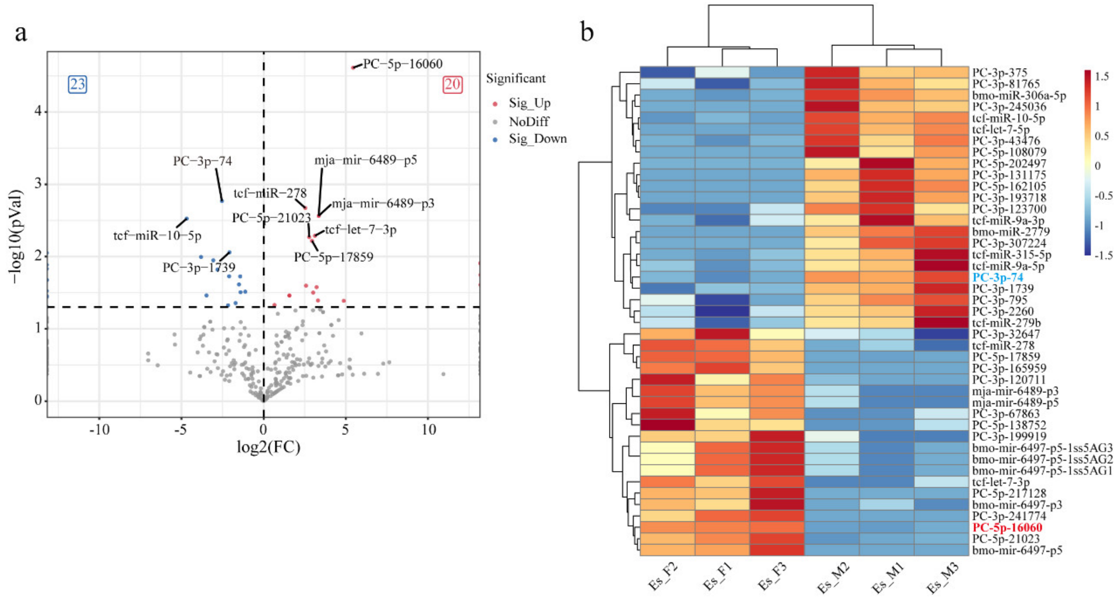

Figure S2. Differential miRNAs (DEMs) expression in *E. sinensis* female and male reproductive systems. (a) The volcano map of DEMs in females versus males. The X-axis represented the log2 (fold change) in the miRNA expression between female and male crabs. In contrast, the statistical significance of changes in miRNA expression was presented at the Y-axis. Individual miRNAs were represented by the scatter pointed in the graph, and the grey dots each indicated no significant difference. The red dots indicated significantly up-regulated differential miRNAs, while the blue dots represented significantly down-regulated differential miRNAs. **(b)** A heat map diagram depicting the differentially expressed miRNAs in the female and male reproductive systems of *E. sinensis*. The color indicates that the *p*-value ranged from high (red) to low (blue).

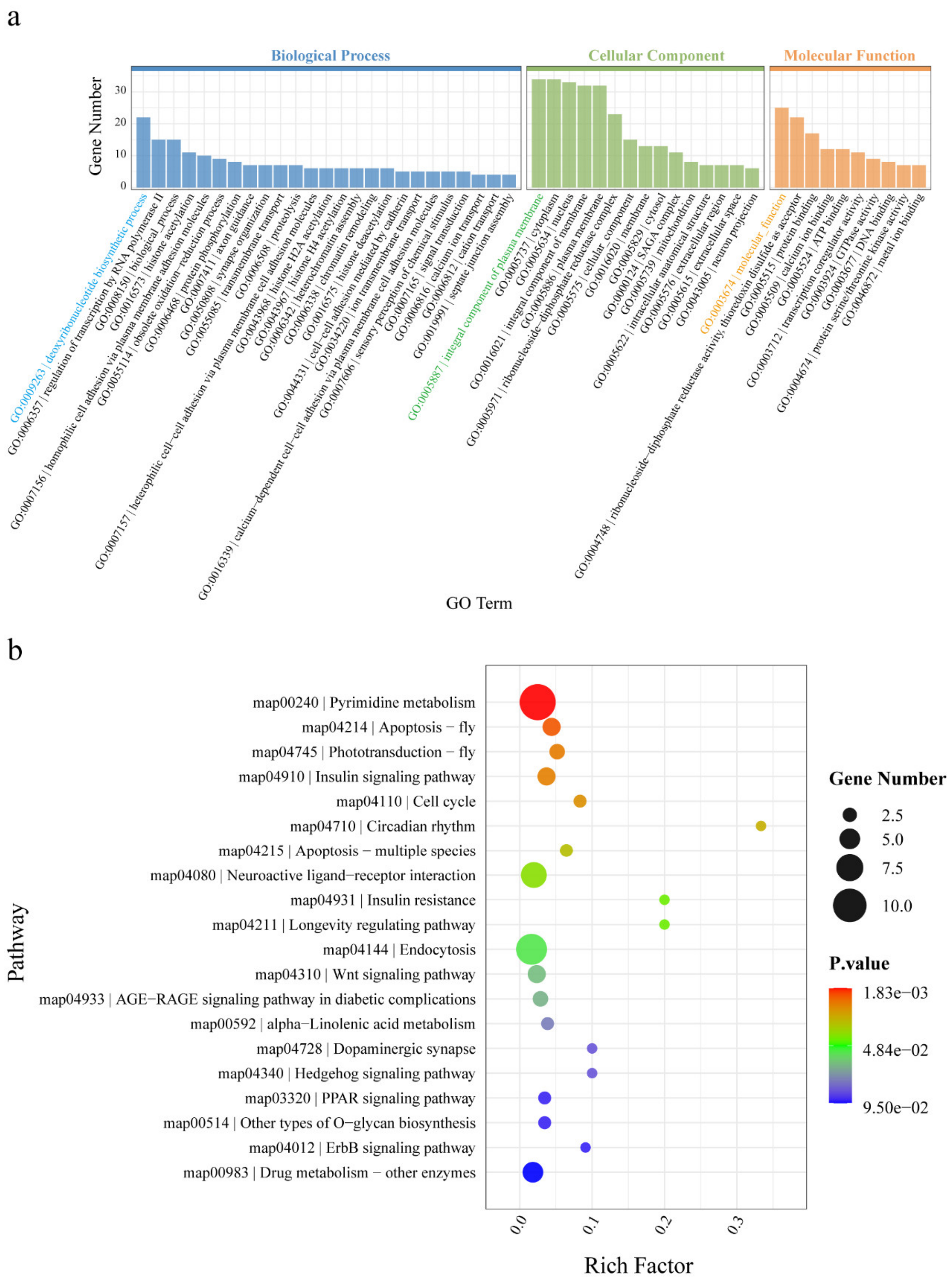

Figure S3. GO and KEGG classification for predicted target genes of DEMs. (a) The GO term for the next level of the three major classes of GO in the abscissa. The number of candidate target genes annotated to the term (including the sub-term of the term) was represented on the Y-axis. **(b)** Summary of KEGG pathway enrichment for predicted target genes of DEMs. The Y-axis represented the name of the pathway, while the X-axis represented the Rich factor. The number of candidate target genes in the pathway was indicated by the size of the point, and the color of the point corresponded to a different *p*-value range.
